# SNP Design from 454 Sequencing of *Podosphaera plantaginis* Transcriptome Reveals a Genetically Diverse Pathogen Metapopulation with High Levels of Mixed-Genotype Infection

**DOI:** 10.1371/journal.pone.0052492

**Published:** 2012-12-27

**Authors:** Charlotte Tollenaere, Hanna Susi, Jussi Nokso-Koivisto, Patrik Koskinen, Ayco Tack, Petri Auvinen, Lars Paulin, Mikko J. Frilander, Rainer Lehtonen, Anna-Liisa Laine

**Affiliations:** 1 Metapopulation Research Group, Department of Biological and Environmental Sciences, University of Helsinki, Helsinki, Finland; 2 Institute of Biotechnology, University of Helsinki, Helsinki, Finland; American University in Cairo, Egypt

## Abstract

**Background:**

Molecular tools may greatly improve our understanding of pathogen evolution and epidemiology but technical constraints have hindered the development of genetic resources for parasites compared to free-living organisms. This study aims at developing molecular tools for *Podosphaera plantaginis*, an obligate fungal pathogen of *Plantago lanceolata.* This interaction has been intensively studied in the Åland archipelago of Finland with epidemiological data collected from over 4,000 host populations annually since year 2001.

**Principal Findings:**

A cDNA library of a pooled sample of fungal conidia was sequenced on the 454 GS-FLX platform. Over 549,411 reads were obtained and annotated into 45,245 contigs. Annotation data was acquired for 65.2% of the assembled sequences. The transcriptome assembly was screened for SNP loci, as well as for functionally important genes (mating-type genes and potential effector proteins). A genotyping assay of 27 SNP loci was designed and tested on 380 infected leaf samples from 80 populations within the Åland archipelago. With this panel we identified 85 multilocus genotypes (MLG) with uneven frequencies across the pathogen metapopulation. Approximately half of the sampled populations contain polymorphism. Our genotyping protocol revealed mixed-genotype infection within a single host leaf to be common. Mixed infection has been proposed as one of the main drivers of pathogen evolution, and hence may be an important process in this pathosystem.

**Significance:**

The developed SNP panel offers exciting research perspectives for future studies in this well-characterized pathosystem. Also, the transcriptome provides an invaluable novel genomic resource for powdery mildews, which cause significant yield losses on commercially important crops annually. Furthermore, the features that render genetic studies in this system a challenge are shared with the majority of obligate parasitic species, and hence our results provide methodological insights from SNP calling to field sampling protocols for a wide range of biological systems.

## Introduction

Molecular tools in parasite species can address a range of exciting questions both from evolutionary and ecological perspectives [1]. There is an urgent need for such tools also from an applied point of view, as parasites impose significant threat on human health and agriculture. To date however, due to scarcity of suitable genetic markers, molecular ecology studies on parasites are lagging far behind those on free-living organisms [2], [3]. The features that make an organism parasitic are the same ones that complicate molecular studies: their small size limits the amount of DNA available and contamination by host DNA may be difficult to avoid, especially in obligate parasites. Furthermore, clonal reproduction, a common feature in parasite species, renders the identification of individuals/strains/genotypes a challenge.

*Podosphaera plantaginis,* formerly *Sphaerotheca plantaginis*, (Erysiphales, Ascomycete, Castagne; U. Braun & S. Takamatsu) is an obligate powdery mildew pathogen of the ribwort plantain *Plantago lanceolata* L. (Plantaginaceae). The occurrence of this pathogen has been intensively studied since 2001 in the Åland archipelago, south-west Finland, with more than 4,000 host plant populations surveyed each year by 40–70 students recording the presence-absence of the pathogen (for details on the large-scale survey, see [4]). The pathogen remains rare (maximum 7% of the patches infected in a given year) and persists as a highly dynamic metapopulation, with frequent extinction and (re)colonisation events of local host populations [4], [5]. During the growing season the epidemic builds up following repeated asexual cycles of reproduction, as the mildew is wind-transmitted within [6] and among [4] host populations. At the end of each growing season the pathogen populations crash as most host individuals die back to root stock. The interaction between *Pl. lanceolata* and *Po. plantaginis* in the Åland archipelago offers unique opportunities for finding links between local evolutionary dynamics and realized epidemiological patterns and has been recognized as one of the model systems for studying disease evolution in wild plant-pathogen interactions [7]. Breakthrough discoveries to date include the short spatial and temporal scales of resistance evolution under pathogen attack [8], [9], as well as the coupling of pathogen evolution with ambient temperature regime [10].

The development of genetic resources for *Po. plantaginis* opens exciting new research directions for this system. Like with many parasitic species capable of clonal reproduction, morphological observation does not allow to distinguish the different strains of *Po. plantaginis* and hence, molecular tools are required for measuring strain diversity in this pathosystem. In particular, the study of mixed-genotype infection patterns (i.e. infection of an individual host by various pathogen strains) can only be elucidated with suitable molecular tools. This is non-trivial, as co-infection is considered to be a key force driving pathogen evolution. Theoretically competition for limited host resources under co-infection is expected to promote more aggressive growth and reproduction of co-occurring strains than under conditions of single infection [11], [12]. As this scenario can lead to non-optimal levels of virulence [13], high relatedness among co-infecting strains is expected to favour less competitive interactions and hence, lower levels of virulence [14], [15]. Overall, understanding evolutionary dynamics under competition is critically important as natural selection can act in contrasting directions depending on the presence or absence of a competitor [14], [16], and as these within host dynamics can have profound implications for between host dynamics, *i.e.* transmission during epidemics [17]. Furthermore, co-infection whereby distinct pathogen genotypes come into close contact is the prerequisite of sexual reproduction for many fungal pathogens, a fundamentally important process for the evolutionary potential of pathogen populations [18]. To date, theoretical advances of mixed-genotype infection have outpaced our understanding of natural systems given the scarcity of suitable molecular tools [1]. In addition to the study of mixed-genotype infection patterns, the design of molecular markers in *Po. plantaginis* also allows the description of the metapopulation genetic structure and the study of molecular epidemiology whereby past disease spread scenarios may be reconstructed and future risks of disease spread assessed through molecular characterization of the pathogen. Other exciting venues of research that open up include study of the genetic structure driving adaptive patterns, as well as the investigation of the reproductive strategy of this plant pathogen.

Powdery mildews are among the most devastating pathogens attacking crops worldwide [19], yet genomic resources for these fungi remain scarce. The genome of *Blumeria graminis* was recently released [20] and genome sequencing is under way for two other species (*Golovinomyces orontii* and *Erysiphe pisi,* Max Planck Institute for Plant Breeding Research, Cologne). Transcriptome sequencing was also performed in *B. graminis* [21], *G. orontii* [22] and *Erysiphe necator* [23]. The powdery mildew group is divided into five major clades [24], [25], that have diverged about 70 Myr [26]. However, none of the above mentioned genetic resources concern the Cystotheceae clade comprising *Po. plantaginis*. This is surprising considering the agronomical importance of this group encompassing very important crop pathogens (infecting apple, rose, strawberry or cucurbits for example [18]). Consequently, this study does not only bring new genetic resources for a fascinating natural plant-pathogen system, but also significantly increases the genomic resources of an important plant pathogenic fungal family.

Single Nucleotide Polymorphism (SNP) markers have become the genetic marker of choice in ecology and evolution as a consequence of their numerous advantages: wide and putatively random distribution in the genome, simple and low cost genotyping allowing high-throughput screening, co-dominant inheritance, high between-lab repeatability and well-defined mutation model [27]–[29]. Neutral SNP markers are highly suitable for population genetics studies [30], whereas other SNP loci may be functionally important and may be involved in the identification of ecologically relevant traits [31]. The use of next-generation sequencing (NGS) tools, such as for example the Roche GS FLX 454 (454 Life Sciences/Roche Applied Biosystems, USA) pyrosequencing technology [32], allows identification of numerous SNP loci [33], [34]. However, for non-model organisms without a reference genome available, a genome reduction step is required to acquire deep assemblies of redundant contigs required for SNP discovery [35] and that would be especially true in our case given the complexity of powdery mildew genomes. Indeed, the genome size of the three sequenced powdery mildew species is estimated to be 120–160 Mb, more than four times larger than the median of other Ascomycetes [20]. On the other hand, the number of genes in the powdery mildew family is particularly low (5 854 genes in *B. graminis*) compared to other fungi. Sequencing the transcriptome instead of the genome appears to be a good strategy to reduce data complexity, while including functionally important genes [34]. Indeed, SNP marker development through NGS of transcriptome data has consequently become more and more common in non-model species over the past few years (see for example [36]–[38]). However, we are not aware of any study of this kind in fungi (but see Broders, et al. 2011 for SNP design through genome sequencing).

Here, we describe a method to develop molecular tools in an obligate plant pathogen that may be used to gain insights into the ecology and evolution of a wild host-pathogen interaction. First, the development of molecular resources included the 454 sequencing of a pooled RNA sample, *de novo* assembly and annotation of the transcriptome data and subsequent identification of SNP loci. It finally led to the design of a set of 27 SNP markers that can be simultaneously genotyped. Although the SNP array designed in this study would mostly be relevant to *Po. plantaginis* in the Åland archipelago because of ascertainment bias and lack of cross-amplification [29], we also describe here some functionally important genes such as mating-type genes (controlling sexual reproduction) and candidate effectors (potentially important in pathogenicity). Hence, the transcriptome sequences represent a wealth of relevant resources for functional genomics studies in *Po. plantaginis* and in the powdery mildew fungi in general. We then use our newly developed molecular tools to estimate levels of mixed-genotype infection at the leaf level, i.e. various pathogen strains occurring on the same host leaf (also referred to as co-infection) in this haploid organism, a phenomenon assumed to be crucial for the evolution of pathogen virulence (see above).

## Materials and Methods

### Fungal RNA Material

**Figure 1:**
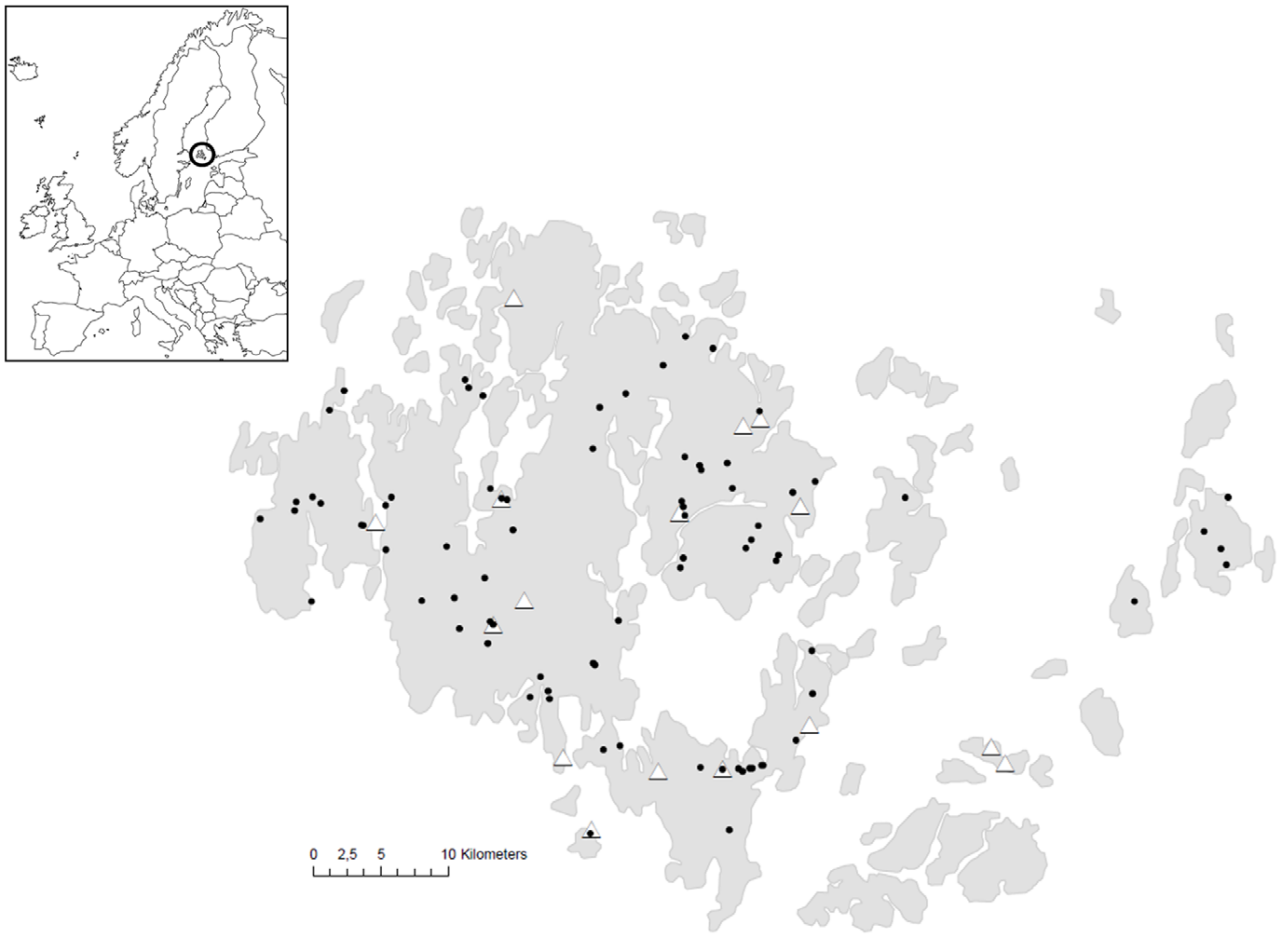
Sampling location within the Åland archipelago. The location of the 16 *Po. plantaginis* sampling sites for the pooled 454 RNA sequencing is represented with white triangles and the location of the 80 sampling sites for the genotyping dataset used for SNP validation is represented with black circles.

The sampling of *Pl. lanceolata* leaves infected with *Po. plantaginis* was performed in September 2008. Neither the host plant nor the pathogen is protected species and Finnish legislation (“*Jokamiehenoikeus*”) allows the sampling of wild species to everyone. The aim was to generate a sample comprising much of the variation present in the pathogen metapopulation in the Åland archipelago, and hence strains from 16 different populations across the archipelago (Figure 1) were pooled into a single sequencing experiment. The samples were collected as infected leaves from the natural populations, and placed on moist filter papers in petri dishes placed in a growth chamber at 20°C. After 5 days the samples had produced fresh conidia and the spores were collected by scraping them off the leaf into 1.5 ml microcentrifuge tubes and samples were then stored in liquid nitrogen.

RNA was extracted as follows: Lysing buffer (100 mM Tris-HCl pH 8.3, 300 mM NaCl, 10 mM EDTA, 0.5% SDS, 400 µg/ml Proteinase K, 20 µg/ml glycogen) was preheated to 65°C. The samples were dissolved to 300 µl of lysis buffer followed by incubation at 65°C for 1 hr with occasional mixing. Large aggregates were removed by centrifugation in Eppendorf centrifuge (13000 rpm, 5 min at room temperature). The supernatants were extracted with an equal volume of hot (80°C) phenol:cloroform:isoamyl alcohol (25∶24:1; pH ∼5). The phenol extraction was repeated 2–3 times, followed by a cloroform:isoamyl alcohol (24∶1) extraction and EtOH precipitation. The pellet was dissolved in 12 µl H_2_O and the RNA concentration was measured.

### 454 Sequencing and Transcriptome Assembly

Normalized cDNA libraries were produced by Evrogen Inc (Moscow, Russia; www.evrogen.com), as described previously [36]. Libraries were further amplified using Illustra GenomiPhi V2 DNA Amplification Kit (GE Healthcare, UK) according to the manufacturer’s recommendations. The libraries were sequenced with the 454 Genome Sequencer FLX using GS FLX series reagents (Roche) at the Institute of Biotechnology (University of Helsinki, Finland). Sequence reads were screened for low quality sequences, sequencing adaptors, normalization and PCR primers with a custom script (J. Nokso-Koivisto, unpublished). Possible chimeras and reads with low qualities were removed from the dataset. Primers and adaptors were masked to lower case characters. De novo assembly was done by MIRA v 3.2.1 [39] in the CSC (IT Center for Science, Helsinki, Finland) Hippu cluster.

### Annotation and Identification of Interesting Genes

**Figure 2:**
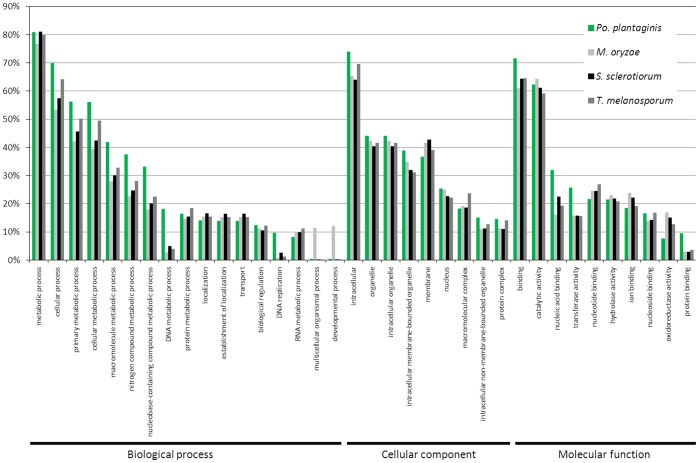
Frequency of the Gene Ontology (GO) terms found in transcriptome sequences of *Podosphaera plantaginis*, compared to the genomic data of three other fungal species: *Magnaporthae oryzae*, *Sclerotinia sclerotiorum* and *Tuber melanosporum*.

The assembled contigs were annotated with descriptions and gene ontology (GO) classes by using the PANNZER tool (Koskinen *et al.*, unpublished). To compare similarity of GO annotations between species we also analysed all the proteins and GO annotations of *Magnaporthae oryzae*, *Sclerotinia sclerotiorum* and *Tuber melanosporum* from UniProt database. GO classes were narrowed down to Protein Information Resource (PIR)-slim GO vocabulary for better comprehension of data. Ratios were calculated as follows: GO classes under e.g. Biological Process category were divided by the total count of Biological Process annotations in the datasets. GO classes under Molecular Function and Cellular Component categories were done in similar manner. The visualization of some representative GO classes from each category is shown in Figure 2.

We performed database searches to determine whether orthologous sequences of interesting genes (mating-type genes, avirulence genes and effectors, see details in the results) could be found in our contigs. We also described a set of candidate secreted effectors proteins (CSEPs), as peptides having a signal peptide (as thus excreted from the cell) but no transmembrane domains (excluding peripheral membrane proteins) and no orthologous sequences outside the powdery mildews [20], [40]. Amino acid sequences containing a signal peptide and transmembrane domains were identified using SignalP 4.0 [41] and TMHMM v. 2.0 [42], respectively, both on the web server http://www.cbs.dtu.dk/services/.

### SNP Discovery

The contigs were screened for possible SNPs with a custom script (J. Nokso-Koivisto, unpublished). The criteria to detect probable polymorphic SNP were: 3× occurrence of a minor allele, 7× minimum total coverage at the position and minor allele frequency more than 20%. To validate that the identified SNPs were highly polymorphic in our setting, we sequenced a subset of field samples collected in 2010 (randomly taken from the sampling described below) using Sanger technique. Primers were designed to amplify a 350–600 bp region flanking the expected SNP site using Primer 3 (Available http://frodo.wi.mit.edu/primer3/). DNA was extracted using E.Z.N.A. plant DNA kit (Omega Biotek, USA) following the manufactureŕs instructions, with the final elution performed in 100 µl. PCR reactions were performed in 20 µl final volume containing 2 µl of DNA, 0.5 µM of each primer, 800 µM of deoxyribonucleotides (dNTPs) and 0.6 U Taq polymerase (DyNAzyme II, Finnzymes, Finland) in appropriate 1x buffer. Samples were subjected to an initial denaturation at 94°C for 2 minutes, followed by 35 cycles of denaturation at 94°C for 30 s, annealing at 54°C for 30 s and extension at 72°C for 20 s, with a final extension step of 10 min at 72°C. PCR products were purified using ExoSAP-IT (GE Healthcare, UK). Sequencing reactions and capillary electrophoreses were performed by the Finnish Institute for Molecular Medicine (FIMM, Helsinki, Finland).

### Design of the SNP Panel and Genotyping

The program MassARRAY Assay Designer (Sequenom, CA, USA) was used to design the SNP panel, which was then validated by genotyping in a global sample collected in the Åland archipelago in September 2010. In that year, a total of 175 *Plantago* patches were infected by the powdery mildew and we collected four or five samples in 80 of these patches (Figure 1), depending on the local population size. Each sample was collected as an entire infected *Plantago* leaf placed in a falcon tube in the field. Samples were then prepared by collecting a 1 cm^2^ piece of infected leaf as well as all fungal material that could be scraped off the leaf with a scalpel into 1.5 ml microcentrifuge tube kept at −80°C. Both plant and fungal DNA were thus extracted jointly at the Institute of Biotechnology, using E.Z.N.A. plant DNA kit as described before. Genotyping was performed using Sequenom iPLEX SNP platform at FIMM. The software Plotter (Wong and Lehtonen, unpublished) was used to visualize the results.

### Estimation of Mixed-genotype Infection Levels

*Podosphaera plantaginis* is a haploid fungus and consequently, one allele per sample is expected in case of single-genotype infection whereas both alleles can eventually be found in case of mixed-genotype infection. To validate such expectations under co-infection, we experimentally produced mixed-genotype infection by mixing nine combinations of two different strains in different proportions (see details in Supplementary Table 1). First, equal amount of each strain was obtained by putting two pieces of infected leaf of approximately the same size in 1.5 ml microcentrifuge tube and DNA was extracted as previously described. Second, 75/25 proportions were obtained by mixing 3 pieces of infected leaves bearing one strain with one bearing the other strain. Finally, we mixed a piece of infected leaf bearing one strain with spores of another strain to obtain the so-called “90/10 proportion”, roughly mimicking the field procedure (i.e. scraping all the spores and mycelia of the entire leaf onto a piece of leaf, see above).

**Table 1:** Characteristics of the 33 SNP loci discovered in *Po. plantaginis populations* from the Åland archipelago: contig number, functional description of the gene (inferred from PANZER) and minor allele frequency in the dataset genotyped (the latter being available only for the 27 loci included in the SNP genotyping set).

	SNP ID	contig ID	Gene description	Freq (minor allele)
**1**	harma_c1217_640	c1217	ARF GTPase activator protein (Csx2); Centaurin beta	0.35
**2**	harma_c1336_788	c1336	KLTH0H14322p	0.32
**3**	harma_c1421_219	c1421	Diphthamide biosynthesis protein	0.26
**4**	harma_c1421_455			0.23
**5**	harma_c1617_437	c1617	Polyamine transporter 2	0.40
**6**	harma_c1617_624			unknown
**7**	harma_c1720_2036	c1720	SH3 and PX domain-containing 3-like protein; Sorting nexin MVP1	0.30
**8**	harma_c1728_2255	c1728	Transcriptional regulator Ngg1	unknown
**9**	harma_c1817_1349	c1817	Aspartic-type endopeptidase (OpsB); Gastricsin	unknown
**10**	harma_c1892_2119	c1892	DNA replication factor C subunit; Chromosome transmission fidelity factor	0.49
**11**	harma_c2006_2561	c2006	COG1587: Uroporphyrinogen-III synthase (ISS)	unknown
**12**	harma_c2493_601	c2493	Mitochondriale tricarboxylate carrier protein; Tfap2a; 2-oxoglutarate dehydrogenase	0.02
**13**	harma_c2493_784			unknown
**14**	harma_c2543_643	c2543	WD-repeat containing protein slp1; Cell division cycle protein Cdc20	0.44
**15**	harma_c26575_623	c26575	AVRa10	0.01
**16**	harma_c2804_701	c2804	Dolichyl pyrophosphate Glc1Man9GlcNAc2 alpha-1,3-glucosyltransferase	0.18
**17**	harma_c3117_1457	c3117	Predicted CDS Pa_1_13970	0.32
**18**	harma_c3926_348	c3926	Developmentally regulated GTP-binding protein 1, putative	0.20
**19**	harma_c3997_508	c3997	Mediator of RNA polymerase II transcriptionsubunit; DNA-directed RNA polymerase III subunit RPC10	0.01
**20**	harma_c4769_1106	c4769	MarY1-like reverse transcriptase (Fragment)	0.35
**21**	harma_c4833_630	c4833	Mitochondrial mRNA processing protein PET127	0.14
**22**	harma_c5096_985	c5096	Mitochondrial membrane protein Pet127	0.47
**23**	harma_c5876_431	c5876	Arg-6; Acetylglutamate kinase 1	0.09
**24**	harma_c6190_286	c6190	Membrane protein, putative	0.20
**25**	harma_c6267_404	c6267	Mitochondrial exoribonuclease Cyt-4, putative	0.50
**26**	harma_rep_c1961_692	rep_c1961	Reverse transcriptase, gag, polyprotein; copia-like polyprotein	0.06
**27**	harma_rep_c465_1592	rep_c465	Predicted CDS Pa_5_3340	0.41
**28**	harma_rep_c542_236	rep_c542	Topisomerase II associated protein (Pat1); Transmembrane lipoprotein	0.40
**29**	harma_rep_c6068_457	rep_c6068	TE2	0.41
**30**	harma_rep_c664_2300	rep_c664	Ubiquitin carboxyl-terminal hydrolase 12	0.10
**31**	harma_rep_c707_1102	rep_c707	Mitochondrial ribosomal protein L23, putative	unknown
**32**	harma_rep_c707_1118			0.42
**33**	harma_rep_c707_1234			0.42

## Results

### Transcriptome Characterization, Annotation and Identification of Some Interesting Genes

We obtained a total of 549,411 sequence reads with average length of 380 nucleotides (nt). 452,711 of the reads exceeded our quality threshold and were assembled into 45,245 contigs of average length 943 nt. The N_50_ contig size was 1063 nt and 78.35% of the contigs were longer than 500 nt (Supplementary Figure 1a). Average sequencing depth was 2.68 reads per nucleotide (median depth of 1.76; Supplementary Figure 1b). The total consensus transcriptome length was 42.69 Mb. Annotation revealed that 82.9% of the sequences belonged to the Eukaryota (12.2% bacteria), among which 70.6% were fungal sequences (5.4% as plant and 6.1% as Metazoan). We were able to annotate 29505 unique contigs (65.2% of the contigs), with 11177 (37.88%) having a unique annotation. 21578 unique descriptions were found in annotations.

The study of potential avirulence genes, as well as effector proteins would be highly interesting in the context of host-pathogen coevolution and we consequently applied four different methods to identify such candidate genes of interest in the *Po. plantaginis* transcriptome sequences. First, we searched for homologs to proteins already described as potentially important for fungal pathogenesis. According to PANZER annotation tool, 83 contigs were described as putative virulence effectors from their homology with virulence effectors described in other fungal species. When searching for homologs of the AVRk1 and AVRa10 sequences (the EKA family) [43], [44] using TBLASTX, we obtained 27 contigs having e-values lower than 10^−5 ^for at least one of the two effectors. Finally, we searched for homologs of the 491 proteins identified as CSEPs in *Blumeria graminis* in our dataset using TBLASTN and obtained 58 hits with e-values lower than 10^−5^. These hits corresponded to 45 different contigs. Second, we attempted to identify new CSEPs from our sequences. From our dataset of 24 323 predicted peptides longer than 70 amino acids, we had 320 peptides having a signal peptide, 251 of them lacking a transmembrane domain and 19 of them having no orthologous sequences except within the powdery mildews. A list of 19 candidate effectors not yet described was thus identified from our dataset. All the contigs identified as coding for potential effectors are listed in the Supplementary File 2 and a Neighbor-joining tree of these contigs is represented in the Supplementary Figure 2. Very few overlaps were found between the four different methods used to identify potential effectors: only three contigs were similar with both proteins of the EKA family and *Blumeria* CSEPs.

The mating-type (MAT) genes were recently described in few Erysiphales species [45]. We searched for the MAT genes of *Erysiphe necator* in our library using TBLASTN and found the following orthologs to all five searched genes: MAT1-1-1 (Genbank Protein Accession number: AEB33762.1 similar to the contig c38374, score = 100 and e-value = 8.10^−22^), MAT1-2-1 (AEB33764.1, c1683, score = 218, e-value = 4.10^−57^), MAT1-1-3 (AEB33763.1, c10509, score = 108, e-value = 4.10^−24^), SLA2 (AEB33761.1, c3339,score = 359, e-value = 7.10^−100^), and APN2 (AEB33765.1, c24344, score = 119, e-value = 2.10^−27^).

### SNP Discovery

Using the criteria previously described, we identified 4200 SNPs in 1806 contigs. The SNP occurrence rate was 0.03 SNP/100 bp over the 14.33 Mb of the 7475 contigs having a maximum coverage of at least seven (seven reads is the minimum coverage needed to observe a SNP according to our criteria). As most of the re-sequenced contigs did not present the expected polymorphism, we finally designed primers for 165 contigs for Sanger sequencing. We obtained high quality sequence for 99 loci (60% of the investigated loci) but only 20 amplified regions presented polymorphism for the expected site (20.2% of the contigs with high quality sequence). While examining the Sanger sequencing results, we found 13 additional polymorphic sites located in eight different contigs (8.1% of the contigs with good sequence). These 13 newly identified SNPs had variable status in the original 454 transcriptome dataset: five of them were monomorphic, five SNPs showed some polymorphism but did not fulfil our minor allele frequency criteria (minimum 3 reads), two SNP sites had too low sequencing depth and finally, one SNP was found in an intron. The SNP occurrence rate over the whole re-sequencing dataset (39.5 kb) was 0.081 SNP/100 bp. Overall, we found 33 polymorphic SNP loci within 27 contigs (see Table 1).

### Design and Validation of the SNP Panel

**Figure 3:**
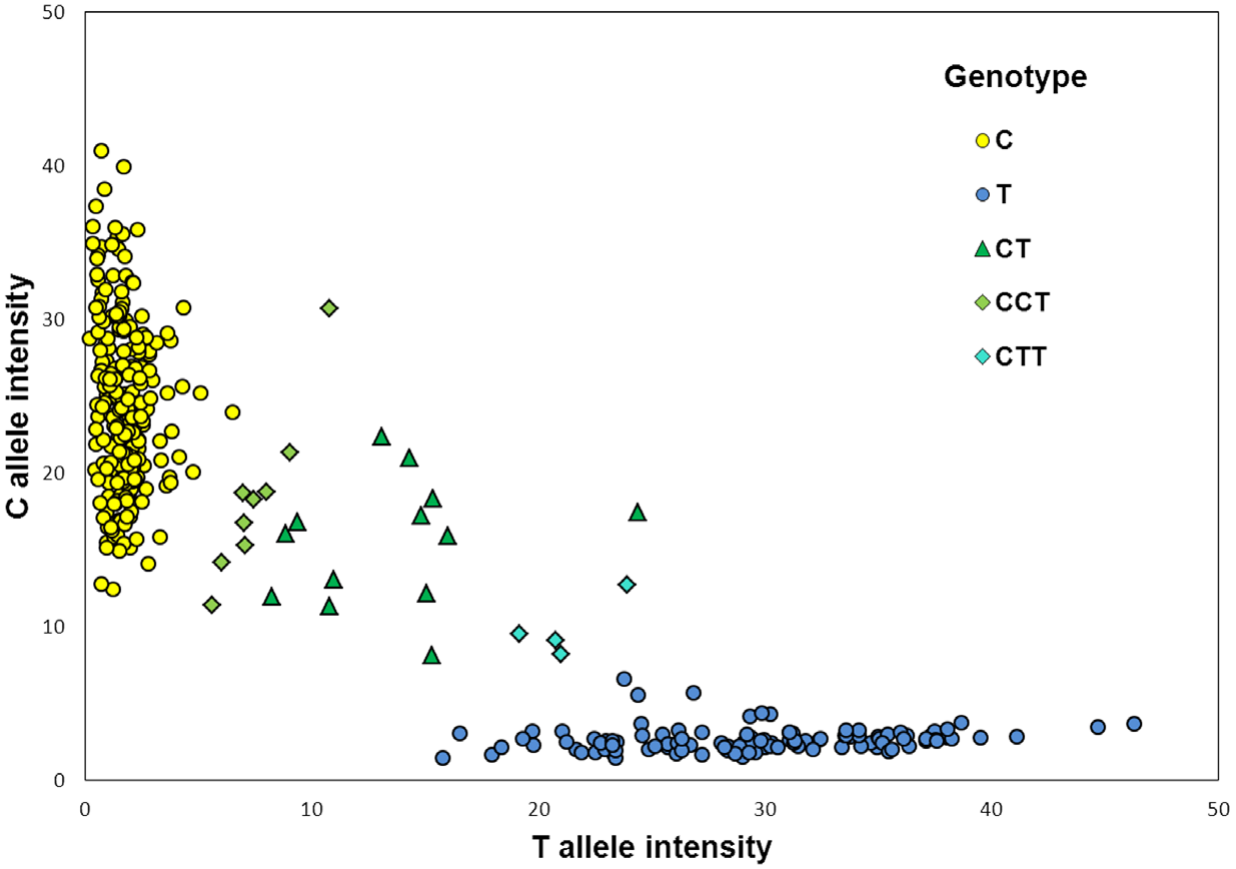
Genotyping result obtained for the locus “harma_c3117_1457” in the dataset of 380 samples (from 80 localities). Single-allele infections are represented with filled circles (C and T genotypes) whereas probable mixed-allele infections are represented with triangles, crosses and stars. CCT and CTT allele combinations were considered respectively as C and T when considering “high level of co-infection” or conservatively as C and T, for analyses mentioned as “low level of co-infection”.

We were able to design a SNP panel including 27 out of the 33 SNP loci (Table 1), among which 25 were located in different contigs, i.e. were apparently unlinked. As expected, as each infected leaf sample may contain one or various *Po. plantaginis* haploid strains, our genotyping produced either only one allele or both alleles for each locus (Figure 3). Our genotyping dataset contained 380 samples in total with 4 or 5 samples per patch (mean = 4.75). The location of the 80 patches is indicated in the Figure 1 (note that only two patches overlapped between the initial sampling for transcriptome sequencing and this SNP genotyping dataset). The overall call rate was 98.8% (127 missing genotypes out of 27*380 = 10260). Maximum number of missing genotypes per sample was 7 only in one sample; 327 samples out of 380 (86%) had no missing value. All the 27 loci were polymorphic in this dataset (Table 1).

### Estimate of Mixed-genotype Infection Levels

As expected, the frequency of samples presenting more than one allele for a given locus (mixed-infection) significantly increased with the frequency of the minor allele (ρ = 0.741 and *p*<0.001 considering high level of co-infection and ρ = 0.689 and *p*<0.001 using low level of co-infection, see Figure 3 legend).

The samples we produced by experimentally mixing two strains in different proportions were detected as presenting both alleles (CT, CTT or TTC pattern, as illustrated in Figure 3) in most of the cases: 96.5% of the cases when the two strains were mixed with equal proportion, 83.5% when proportion was 75/25 and 82.3% when proportion was 90/10 (see more details in Supplementary Table 1). We expected a pattern of decrease of the relative intensity of the allele of one strain when the proportion of the other strain increases. Although the proportion of the two strains in the mix was estimated roughly, such a tendency was generally found (Supplementary Figure 3).

When considering as coinfected the samples with only one locus with two alleles, and less clear genotyping result (for example CCT and TTC for the locus c3117, see Figure 3), the number of samples presenting mixed-genotype infection was 110 (28.9% of the dataset). When applying a more conservative method (considering only samples with at least two loci with two alleles, and clear genotyping result), we still found 48 mixed-genotype samples (12.6% of the dataset).

Out of the 80 mildew populations, 44 (55.0%) contained no genetic diversity at all: only one multilocus genotype (MLG) was detected (we considered diversity if clearly assigned allelic variation was found, or co-infection at least in two samples for at least one locus or co-infection for only one sample but for at least three loci). When considering only the patches presenting some diversity, the proportion of leaf samples with mixed-genotype infection within a patch varied between 0 and 1 (average: 40.0%; samples were then considered as mixed-genotype infection if at least only one locus exhibited a clearly mixed profile, for example CT in Figure 3).

### Genetic Diversity in the Pathogen Metapopulation

**Figure 4:**
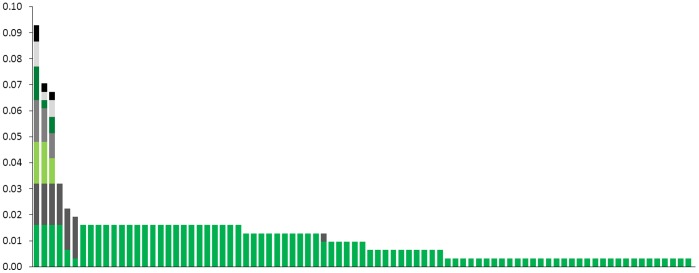
Distribution of the 85 mutilocus genotypes (MLG) found in the whole metapopulation. Each bar represents one MLG, ordered by their frequencies. For each bar the different colours indicate in how many patches the MLG was found.

After having excluded the mixed-genotype samples (defined with the conservative way “low level of co-infection”), we obtained a total number of 85 MLG, with an uneven distribution across the metapopulation (Figure 4). Vast majority of the MLGs (78/85, 91.8%) were found in only one patch whereas four MLGs were found in two different patches (located 0.3 to 35.6 kms away from each other) and three were found in seven patches (with maximum distance of 46.3 kms for the same MLG).

## Discussion

Our 454 sequencing of the *Po. plantaginis* transcriptome, and subsequently developed SNP panel, reveal genetic diversity in the pathogen metapopulation and a high frequency of co-infection. The developed SNP panel proved powerful for detecting genetic variation in this pathosystem, and to date we’ve uncovered other genetic resources that may be important tools for studying the evolution and epidemiology of this system, including virulence effectors and mating type genes. Given the vast information on the epidemiology of this pathogen – recorded since 2001 in over 4000 host populations annually – this holds promise for a fascinating model system for molecular epidemiology studies.

### Transcriptome Sequencing

The project was initiated by 454 sequencing of a pool of RNA samples originating from various location within the Åland archipelago. Obtained sequences were assembled into 45245 contigs, with median length of 1063 nucleotides and median coverage of 1.76. Taxonomic annotation showed that 58.5% of our sequences are homologous to previously described fungal proteins. We cannot expect to obtain an exhaustive representation of the proteins from our species in this low-coverage assembly, as some proteins may be missing or truncated. Moreover, RNA was extracted from scraped material from infected leaves and thus likely contains mostly conidia and mycelia, and little haustoria or cleistothecia. However, functional analysis shows that the distribution of functional annotation is similar to other fungal species (Figure 2). This indicates our assembly encompasses most of the gene functions and consequently represents an unbiased fraction of the total transcriptome of *Po. plantaginis*.

Mating-type genes were found, facilitating projects aiming at a better understanding of the sexual stage of this species. We also identified candidates for proteins involved in pathogenicity, with 145 orthologous proteins to already described fungal effectors and 19 newly described candidate effectors. The list of potential effectors obtained from this study is likely not to represent an exhaustive view of the possible avirulence genes of *Po. plantaginis*; *s*uch exhaustive view could only be obtained from much deeper sequencing of cDNA from various tissues, especially haustoria [22], and various infection stages. Consequently we obtained much less candidates than the 248 effector candidate proteins identified in the genome of *B. graminis* [20] and the 70 found from sequencing haustorial RNA in *G. orontii* [22]. However, not having an exhaustive list of potential effectors does not remove the interest of the identified proteins. The 19 newly described ones appear particularly promising as most effectors are thought to be species-specific [20]. This study may thus allow the initiation of population genomics projects, towards understanding of the molecular basis of the coevolution [46] between this fungal pathogen and its host.

### Single Nucleotide Polymorphism Discovery

The SNP discovery process revealed to be more difficult than expected; indeed, as much as 83% of the 4200 SNPs discovered initially were shown to be monomorphic through Sanger re-sequencing. Previous SNP discovery though NGS attempts have reported highly variable frequency of SNP validation, with for example 48% of the SNPs validated in the rainbow trout, [47], 70% in the coral [37], 83% in Eucalyptus [38] and 85% in maize [48]. Most probable sources of errors include sequencing errors, contaminations from other species, alignment errors (such as the alignment of paralogs [28]), biased sample pooling and sequencing library construction. Alignment errors are likely to have occurred in our case. Indeed, when genotyping 23 loci for 367 individuals in a first SNP panel (data not shown), all the samples presented both alleles for four loci out of 11 polymorphic loci (36.4%), and these four loci were located within regions presenting also additional flanking SNPs. Each of these contigs may thus correspond to various paralog sequences instead of a unique gene.

The use of a pooled RNA sample originating from 16 patches widely distributed within Åland archipelago should avoid the ascertainment bias problem [28]. However, various reasons related to the initial sample may also have contributed to explain the low fraction of confirmed SNPs. First, the pooled RNA sample may have been biased, with some rare genotypes much more represented than others (unequal contributions of individuals [28]). Two samples from Föglö island (extreme South-East of the studied region, Figure 1) were included in the RNA pool but no sample from this area was included for the SNP validation; thus, although unlikely, we cannot rule out the hypothesis that an overrepresentation of the supposedly genetically differentiated Föglö would have contributed to the low validation rate of the SNP loci. Second, temporal changes in the allelic frequencies within the archipelago may have occurred between the initial sampling in 2008 and the sampling used to confirm the SNPs in 2010. Metapopulation dynamics are particularly prone to rapid temporal changes as extinction/recolonisation patterns may lead to the complete reshuffling of the patch genetic contents [49] (see for example [50]).

Overall *Po. plantaginis* genetic diversity within Åland archipelago is likely to be low. Indeed, we found a SNP occurrence rate first of 0.03 SNP/100 bp in the assembly, but this estimate includes non-validated SNPs and 0.081 SNP/100 bp within the re-sequenced regions, which are expected to be biased towards polymorphism as we sequenced regions expected to be polymorphic. Previous studies reported SNP rates ranging between 0.33/100 bp [48] and 0.72/100 bp [51], but the study areas were always far larger than the 50×50 kms of the Åland archipelago. No similar data (SNP detection rate for fungal pathogens at small geographic scale) are yet available for comparisons with the diversity found in our system, as most population genetics studies in fungal pathogens to date have been using microsatellite markers (see for example [52], [53]). However, SNP loci may perform better than microsatellites in a model system exhibiting low levels of diversity [54] and consequently may be the best markers in our case. While the SNPs designed in this study should be applied with caution for samples from other geographic localities than the Åland archipelago, the transcriptome sequences nonetheless provide genetic resources to develop other type of molecular markers such as polymorphic sequences.

### Insights from the Genotyping Data and Perspectives

We tested our SNP genotyping panel in a dataset of 380 samples originating from 80 patches (4–5 samples per patch) throughout the Åland archipelago. The genotyping method was very efficient (98.8% call rate). Extracting both plant and pathogen DNA into the same sample appears thus as a successful strategy: increased DNA amount by including plant DNA likely increased the DNA recovery and therefore the downstream amplification success. Furthermore, SNP markers were specific enough so that the plant DNA did not affect the SNP marker amplification. The practical implications of this cannot be understated, as it enables large-scale field sampling to be carried out when the time consuming purification of samples is not required. Approximately half of the sampling sites presented no diversity at all, with only one multilocus genotype (MLG) found in all four or five samples analysed per site. More intensive sampling was performed for a subset of the patches (up to 30 infected leaves collected) and, although less frequent than in this study, absence of diversity within a patch was also commonly found (about 20% of the analysed patches, Susi & Laine, unpublished data). Lack of diversity is thus not only an artefact resulting from the low within-patch sampling effort of this study. Lack of genetic variability in some patches is likely to prevent the pathogen populatiońs evolution [55] and such differences in genetic diversity may partly explain variation in the pathogen’s capacity to adapt to the local hosts population [8].

Our genotyping method allowed the detection of mixed-genotype infection at the leaf level as, even in the case of unequal mixing of two strains mimicking the field sampling procedure, both alleles were detected in 82.3% of the samples. Mixed-genotype infection appears to be quite common in this system. Indeed, even when applying a conservative detection method, 40% of the samples were mixed-infections in those patches that contain polymorphism. Co-infection levels vary greatly between patches as some patches presenting various MLGs contain no mixed-genotype samples while in some patches all the samples represented co-infection. The few available studies that have investigated mixed genotype infections have detected up to 70% of infections containing more than one pathogen strain (*Microbotryum violaceum* [56], *Mycosphaerella graminicola* [57], *Teratosphaeria nubilosa* [58]). However, the epidemiological and evolutionary consequences of mixed genotype infection have rarely been studied (but see [59]). This phenomenon is however of great importance as it has been suggested to be one of the key forces driving pathogen evolution [60], [61]. Further investigations in our system will assess the effects of mixed genotype infection to pathogen population dynamics and evolution of virulence under natural conditions.

Excluding mixed-genotype infections, we found 85 different MLGs in the whole archipelago. The large majority of them were rare (1–5 samples) and restricted to only one patch. The distribution was highly uneven, with three MLG each representing 5–10% of the samples and found in seven patches, some of them being far away from each other. This wide distribution of a few MLG likely reflects past stepping-stone dispersal. In accordance with previous experimental findings of differences in infectivity between strains, these MLG may then have been maintained through natural selection. This hypothesis could be tested by assessing the link between the genotype and the phenotype through experimental infections of collected samples.

This set of 27 SNP loci consequently opens new research perspectives in this system for which epidemiological data have been collected over 10 years. Our current validation of this SNP panel provides valuable guidelines for sampling intensity for future studies. Combining population genetics and disease surveys into the molecular epidemiology approach [1] is likely to offer much insight into the dynamics and evolution of pathogen populations, both crucial for an efficient control of both human and crop diseases.

## Supporting Information

Figure S1 **Distribution of (1a) the contig length in base pair (bp) and (1b) the average coverage over the contig.** Each histogram bar is divided into annotated contigs in white and unannotated contigs in grey.
(TIF)

Figure S2 **Neighbor-joining tree of the contigs identified as coding potential effector proteins.**
(TIF)

Figure S3 **Genotyping results for the experimental mixed-genotype samples.** Each graph correspond to a particular combination of two different strains (A to I, see also Supplementary Table 1). In every graph, the relative intensity of the allele of the first strain is plotted against the proportion of the second strain in the mix. Each curve corresponds to a particular locus, being polymorphic between the two strains considered.
(TIF)

Table S1 **Results of the genotyping of the experimental mixed-genotype samples.**
(DOCX)

File S1 **Sequence of the 33 SNPs found from the transcriptome sequences of** ***Po. Plantaginis.***
(FAS)

File S2 **List of the contigs identified as coding potential effector proteins.**
(XLSX)
