# Supplementary figures and images for: SNP Design from 454 Sequencing of Podosphaera plantaginis Transcriptome Reveals a Genetically Diverse Pathogen Metapopulation with High Levels of Mixed-Genotype Infection
Source: PLoS One. 2012 Dec 27;7(12):e52492. doi: 10.1371/journal.pone.0052492 (PMC3531457; doi:10.1371/journal.pone.0052492)

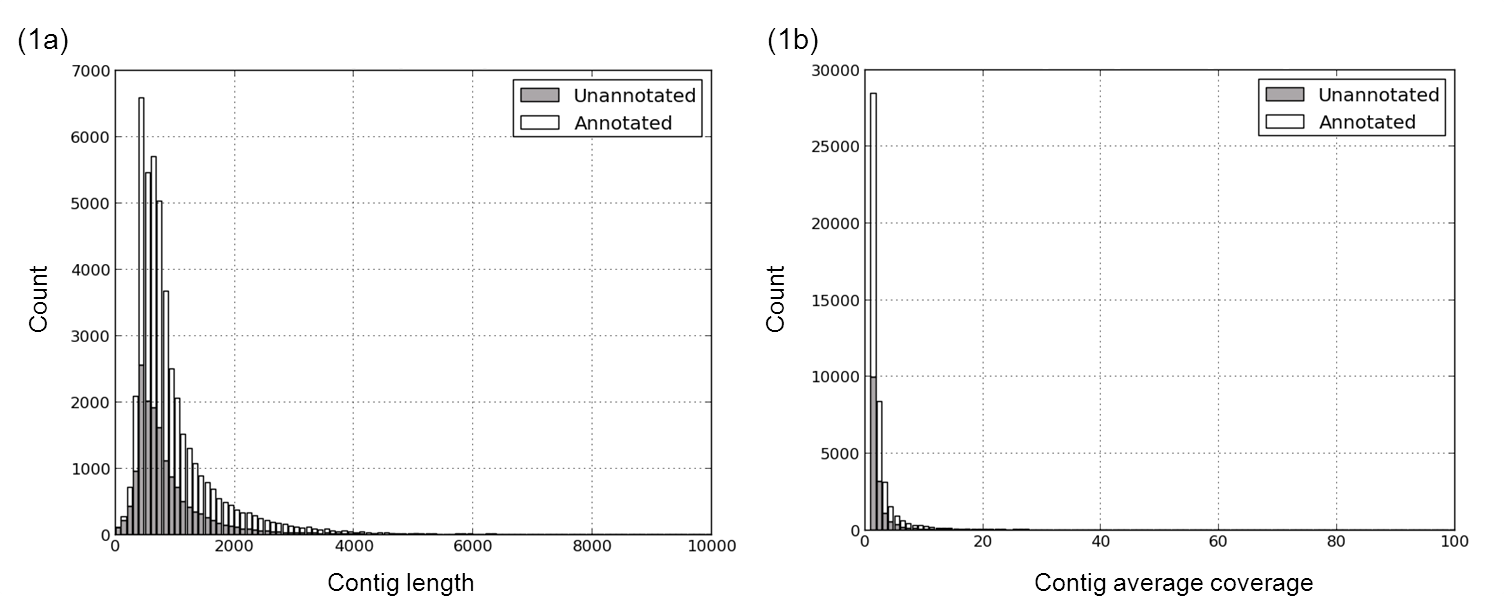

Supplement: Figure S1 — Distribution of (1a) the contig length in base pair (bp) and (1b) the average coverage over the contig. Each histogram bar is divided into annotated contigs in white and unannotated contigs in grey. (TIF) [file pone.0052492.s001.tif]

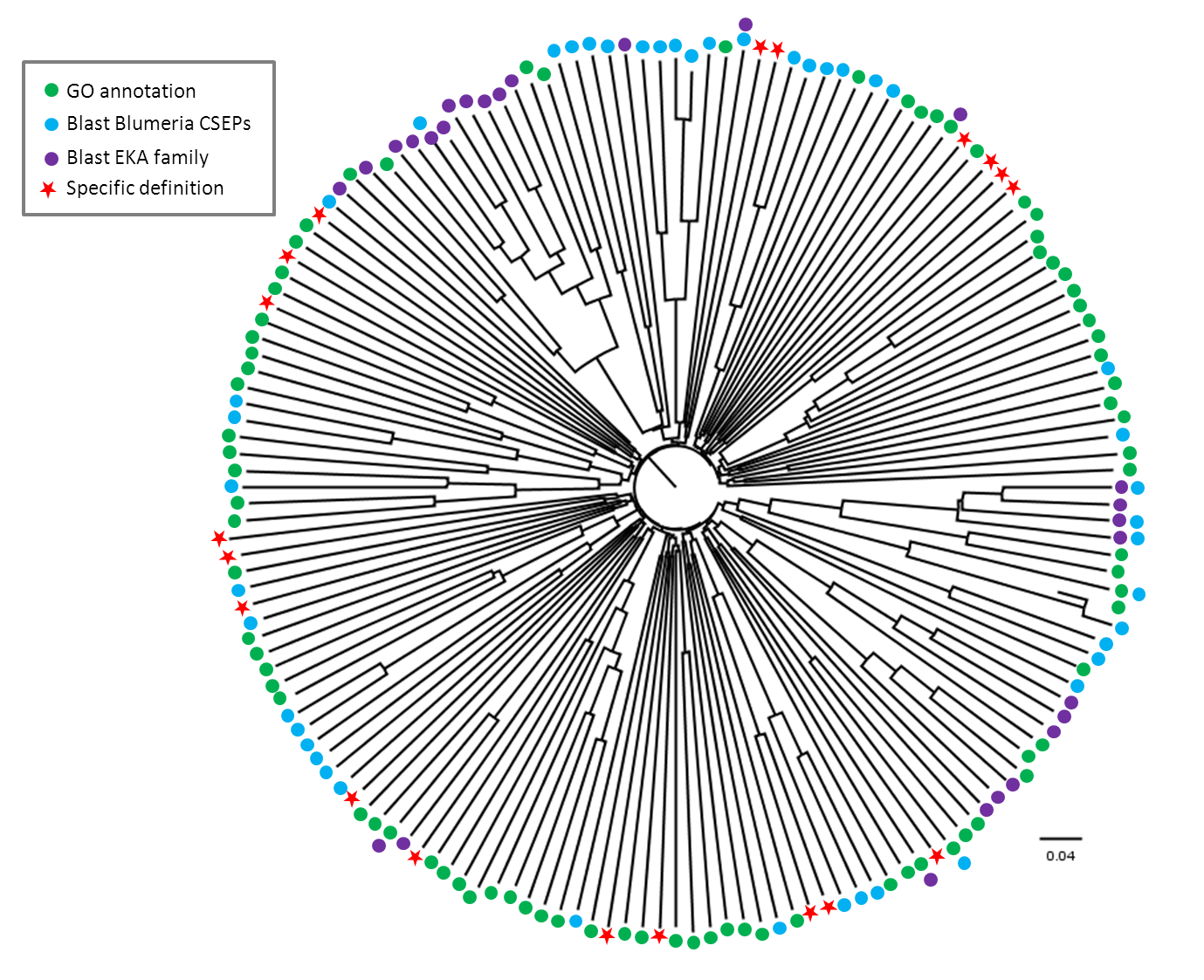

Supplement: Figure S2 — Neighbor-joining tree of the contigs identified as coding potential effector proteins. (TIF) [file pone.0052492.s002.tif]

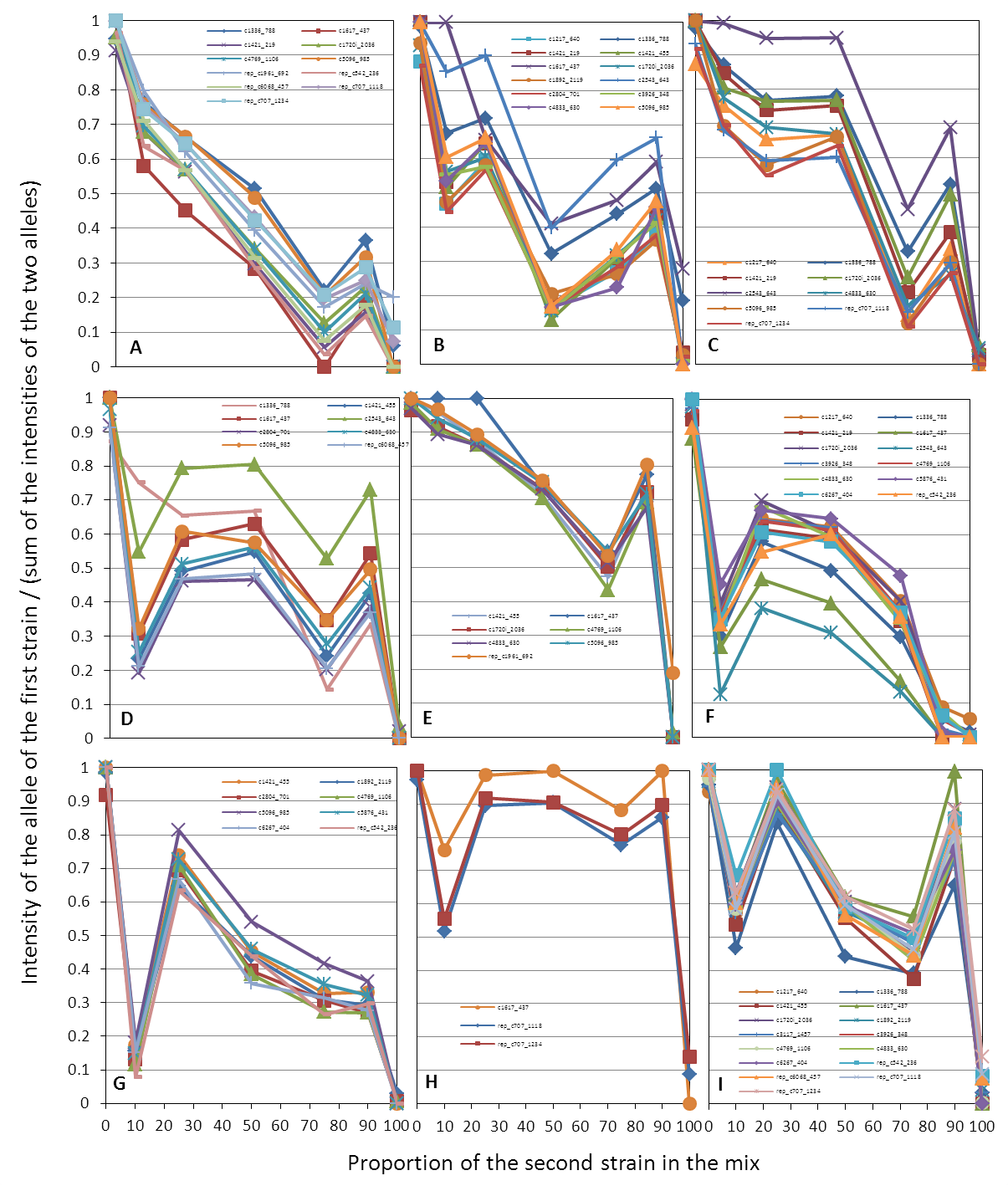

Supplement: Figure S3 — Genotyping results for the experimental mixed-genotype samples. Each graph correspond to a particular combination of two different strains (A to I, see also Supplementary Table 1). In every graph, the relative intensity of the allele of the first strain is plotted against the proportion of the second strain in the mix. Each curve corresponds to a particular locus, being polymorphic between the two strains considered. (TIF) [file pone.0052492.s003.tif]
