# Supplementary material for: SNP Design from 454 Sequencing of Podosphaera plantaginis Transcriptome Reveals a Genetically Diverse Pathogen Metapopulation with High Levels of Mixed-Genotype Infection
Source: PLoS One. 2012 Dec 27;7(12):e52492. doi: 10.1371/journal.pone.0052492 (PMC3531457; doi:10.1371/journal.pone.0052492)
Supplement: Table S1 — Results of the genotyping of the experimental mixed-genotype samples. (DOCX) [file pone.0052492.s004.docx]

Table S1: Results of the genotyping of the experimental mixed-genotype samples.

The number and proportion of success (both alleles detected) is showed for each combination of two different strains and each mixing proportion.

| **Proportions in the strain mix** | | **50/50** | **75/25** | **90/10** |
| --- | --- | --- | --- | --- |
| **Strain combination** | Number of polymorphic loci | Number of loci showing the expected two alleles  (proportion over the number of polymorphic loci) | | |
| **A**  **(1739/1204)** | 11 | 11  (100%) | 7  (63.64%) | 11  (100%) |
| **B**  **(1661/1761)** | 12 | 12  (100%) | 12  (100%) | 11.5  (95.83%) |
| **C**  **(1786/853)** | 9 | 9  (100%) | 8  (88.89%) | 8.5  (94.44%) |
| **D**  **(1271/3030)** | 8 | 8  (100%) | 8  (100%) | 8  (100%) |
| **E**  **(751/900)** | 7 | 7  (100%) | 6.5  (92.86%) | 4  (57.14%) |
| **F**  **(1391/885)** | 12 | 12  (100%) | 12  (100%) | 6  (50%) |
| **G**  **(1602/1786)** | 8 | 8  (100%) | 8  (100%) | 6.5  (81.25%) |
| **H**  **(1602/863)** | 3 | 0  (0%) | 1.5  (50%) | 1.5  (50%) |
| **I**  **(1739/1296)** | 15 | 15  (100%) | 8  (53.33%) | 7  (46.67%) |
| **9**  **(1739/1204)** | 11 | 11  (100%) | 7  (63.64%) | 11  (100%) |
| **All combinations** | 9.44 | 9.11  (96.5 %) | 7.89  (83.5 %) | 7.78  (82.3 %) |
